# Abnormal development of early auditory processing in 22q11.2 Deletion Syndrome

**DOI:** 10.1038/s41398-019-0473-y

**Published:** 2019-04-16

**Authors:** Lucia-Manuela Cantonas, Miralena I. Tomescu, Marjan Biria, Reem K. Jan, Maude Schneider, Stephan Eliez, Tonia A. Rihs, Christoph M. Michel

**Affiliations:** 1 Functional Brain Mapping Laboratory, Department of Basic Neurosciences, University of Geneva, Geneva, Switzerland; 2 Developmental Imaging and Psychopathology Lab, Department of Psychiatry, University of Geneva, Geneva, Switzerland; 3 EEG Brain Mapping Core, Center for Biomedical Imaging (CIBM) of Lausanne and Geneva, Geneva, Switzerland

## Abstract

The 22q11.2 Deletion Syndrome (22q11.2 DS) is one of the highest genetic risk factors for the development of schizophrenia spectrum disorders. In schizophrenia, reduced amplitude of the frequency mismatch negativity (fMMN) has been proposed as a promising neurophysiological marker for progressive brain pathology. In this longitudinal study in 22q11.2 DS, we investigate the progression of fMMN between childhood and adolescence, a vulnerable period for brain maturation. We measured evoked potentials to auditory oddball stimuli in the same sample of 16 patients with 22q11.2 DS and 14 age-matched controls in childhood and adolescence. In addition, we cross-sectionally compared an increased sample of 51 participants with 22q11.2 DS and 50 controls divided into two groups (8–14 and 14–20 years). The reported results are obtained using the fMMN difference waveforms. In the longitudinal design, the 22q11.2 deletion carriers exhibit a significant reduction in amplitude and a change in topographic patterns of the mismatch negativity response from childhood to adolescence. The same effect, reduced mismatch amplitude in adolescence, while preserved during childhood, is observed in the cross-sectional study. These results point towards functional changes within the brain network responsible for the fMMN. In addition, the adolescents with 22q11.2 DS displayed a significant increase in amplitude over central electrodes during the auditory N1 component. No such differences, reduced mismatch response nor increased N1, were observed in the typically developing group. These findings suggest different developmental trajectories of early auditory sensory processing in 22q11.2 DS and functional changes that emerge during the critical period of increased risk for schizophrenia spectrum disorders.

## Introduction

The 22q11.2 Deletion Syndrome (22q11.2 DS; also identified as velo-cardio-facial or DiGeorge Syndrome) is one of the highest genetic risk factors for the development of psychotic disorders^1–3^. Nearly 24% of the adolescents and 30–40% of the adults with 22q11.2 DS develop schizophrenia^4–6^, and up to 60% of the deletion carriers experience subthreshold psychotic symptoms^5,7^.

The 22q11.2 Deletion Syndrome is a multisystem syndrome caused by an interstitial microdeletion of 1.5–3 megabases located on the long arm (q) of chromosome 22, which affects 1 in 4000 live births^8,9^. Notably, it implicates the deletion of 35–60 known genes, many being critical for normal brain development^2,10^.

Although many studies of 22q11.2 DS describe impairments in higher-order cognitive processes such as working memory and executive function^11,12^, there is evidence that, in this disorder, deficits are also manifest at early stages of sensory processing during both visual and auditory tasks^13,14^.

Previous investigations on humans and animal models provide evidence that 22q11.2 deletion syndrome is a valuable neurodevelopmental model to study the functional brain alterations related to schizophrenia^1,15–17^.

Neurophysiological measures, such as event-related potentials have provided robust evidence for both sensory and cognitive dysfunction in schizophrenia and consequently have been proposed as endophenotypes of this psychiatric illness^18^. Two main characteristics are making these measures well suited to study psychiatric illness. First, they can be recorded in passive paradigms, which is an advantage in a population that may be difficult to engage in cognitive tasks. Second, because of their high temporal resolution, neurophysiological measures can be used to study the information flow from sensory to association brain regions, and to determine the stage at which information processing is impaired.

In the present study, we are investigating auditory-evoked potentials (AEPs) elicited by simple auditory stimuli and we focus on the auditory mismatch negativity (MMN) response.

The auditory MMN is an automatic cerebral process that occurs in response to a regularity violation, with or without paying attention, and indexes a prediction error signal^19–22^. Sensory and auditory oddball detection, which is observed in MMN paradigms is critical for everyday function since it reflects the outcome of a survey process that constantly monitors the environment for potentially relevant information. It is generated in the auditory cortex and spreads to additional structures such as the insula, the anterior cingulate cortex and the inferior frontal cortex, leading to bottom-up attentional capture^23–29^.

The MMN is usually elicited in an auditory oddball-paradigm, and it becomes visible after subtracting the response to the frequent stimulus (the standard) from the response to the rare stimulus (the deviant). In the difference waveform (deviant-standard), the MMN can be described as a negative shift, with a typical voltage distribution on the scalp: negativity over the fronto-central channels and positivity over the posterior channels, generally measured between 150–250 ms post-stimulation^30^.

The auditory MMN response is reduced in patients with schizophrenia and in subjects who are at-risk for the development of schizophrenia^31,32^. This effect has been robustly reproduced since the early 1990s^33–35^ and the most reliable deficits are in response to frequency and duration deviants^36^. It has been hypothesized that a reduced duration MMN (dMMN) may index a trait marker of schizophrenia, whereas a reduction in the amplitude of the frequency MMN (fMMN) may be related to lower functioning and progressive brain pathology related to the disorder^37^.

Several mechanistic explanations for a reduced fMMN response in patients with schizophrenia have been proposed. First, bilateral grey matter reduction in Heschl’s gyrus^38^ along with morphological changes of pyramidal cells in layer 3 of the auditory cortex^39^ may have an important role. Second, alterations in glutamatergic neurotransmission may also play a part. The glutamatergic model^40^ of MMN impairments in schizophrenia is based on human and animal studies showing the ability of the NMDAr antagonists, like ketamine and phencyclidine, to reduce the MMN response^41,42^ and further, the ability of the NMDAr agonists, like d-serine, to restore the MMN response^43,44^. These results go in line with histological studies that report hypofunction of NMDAr in patients with schizophrenia^45^.

The characteristics of MMN in 22q11.2 DS are not well understood, as the literature to date is scant and characterized by small sample sizes^46–48^. These cross-sectional studies report no significant decrease in fMMN amplitude at the frontal electrode (Fz) for adolescents and young adults with 22q11.2 DS compared to typically developing participants^46–48^, but a reduced MMN for the duration deviant^48^, and a long stimulus onset asynchrony (>1000 ms), indicating a more rapid decay of the auditory sensory memory trace in 22q11.2 DS^49^.

Concerning the development of the MMN in typically developing individuals, some studies reported increases^50,51^, whereas others reported decreases^52^ or no change^53^ of the amplitude of the mismatch response from childhood to adolescence, even though is well established that the MMN is decreasing in amplitude from young adulthood to older ages^54^.

In recent studies, the auditory MMN has been an informative neurophysiological tool that reflects functional brain changes prior to the emergence of schizophrenia^55^. Therefore, the aim of our study was to investigate the auditory MMN response between 22q11.2 deletion carriers and typically developing individuals before and during a vulnerable developmental window, namely childhood and adolescence. We examined how the MMN response is maturing with age using a longitudinal design. To our knowledge, this is the first study to longitudinally investigate the mismatch response in 22q11.2 deletion carriers. No a priori assumption was made about the changes in mismatch response between childhood and adolescence.

Further, we examined the MMN responses between children and adolescents with 22q11.2 DS and age-matched typically developing individuals in a larger sample using a cross-sectional design. We hypothesized that the auditory mismatch response is reduced in 22q11.2 deletion carriers compared to typically developing individuals.

## Methods

### Participants

The data acquired for this study are part of a longitudinal project of the Swiss National Centre for Competence in Research NCCR Synapsy on 22q11.2 Deletion Syndrome. The participants with 22q11.2 DS were recruited through advertisements in patient association newsletters, while the typically developing individuals were recruited among the siblings of the participants with 22q11.2 DS, and through the local school system. All participants and their parents provided written informed consent according to the protocols approved by the Ethical Committee of the University of Geneva, Switzerland. Prior to inclusion, the presence of the de novo 22q11.2 microdeletion was confirmed using quantitative fluorescence polymerase chain reaction (QF-PCR).

The longitudinal study consisted of 16 participants with confirmed 22q11.2 deletion and 14 typically developing (TD) participants (for demographics see Table 1). The AEPs were measured at two time points (T1—age range 8–14 years and T2—age range 13–19 years).

**Table 1:** Summary of data for demographical and clinical data (longitudinal study)

	TD participants T1 (*N* = 14)	TD participants T2 (*N* = 14)	Carriers T1 (*N* = 16)	22q11.2DS carriers T2 (*N* = 16)
Age (mean age ± s.d.)	12.1 ± 1.3	15.6 ± 1.6	11.4 ± 1.9	15.3 ± 1.9
Gender (M/F)	8/6		12/4	
Full-scale IQ (mean ± s.d.)^a^	111.6 ± 16.2	110.5 ± 14.2	74.8 ± 8.7	75.9 ± 12.8
DICA (*N*)	NA	NA	ADHD (7), Phobia (2), GAD (5), Encopresis (3), ODD (2), MDD (1)	ADHD (8), Phobia (3), GAD (4), MDD (1)
Antipsychotic treatment (*N*)	NA	NA	1	0
Antidepressant treatment (*N*)			0	2
*Methylphenidate* (*N*)			5	2
*SIPS (mean* *±* *s.d.; range)*				
Positive	NA	NA	0.9 ± 1.4; 0–6	0.6 ± 1.1; 0–5
Negative	NA	NA	1.5 ± 1.3; 0–5	2 ± 1.2; 0–4
Disorganization	NA	NA	0.8 ± 1.2; 0–4	1 ± 1.2; 0–4
Generalized	NA	NA	0.8 ± 1.2; 0–5	0.9 ± 1; 0–4

*TD* typically developing, *DICA* Diagnostic Interview for Children and Adolescents, *ADHD* attention deficit hyperactivity disorder, *ODD* oppositional defiant disorder, *MDD* major depressive disorder, *GAD* generalized anxiety disorder

^a^The full scale IQ did not significantly differ in the 22q11.2 deletion carriers (*t* = −0.5, d.f. = 14, *p*-value = 0.5) or the typically developing group (*t* = −1.1, d.f. = 10, *p*-value = 0.2) between T1 and T2

The cross-sectional study included an increased sample of 51 participants with confirmed 22q11.2 deletion, divided into two groups: children (age range 8–14 years old) and adolescents (age range 14–20 years old), and 50 typically developing participants matched for age and gender (for demographics see Table 2). A post-hoc power analysis using Statistica Software indicated that a total sample of 16 participants with 22q11.2 DS for the longitudinal study is enough to detect a large effect size (0.85) with 89% power using paired *t*-tests (*α* < 0.05). Additionally, a total sample of 30 TD adolescents and 31 adolescents with 22q11.2 DS is enough to detect a medium effect size (0.75) with 82% power using unpaired *t*-tests (*α* < 0.05) in the cross-sectional study.

**Table 2:** Summary of data for demographical and clinical data (cross-sectional study)

	TD participants children (*N* = 20)	TD participants adolescents(*N* = 30)	22q11.2DS children(*N* = 20)	22q11.2DS adolescents (*N* = 31)
Age (mean age ± s.d.)	10.4 ± 1.6	15.9 ± 1.6	10.8 ± 1.6	16.9 ± 1.9
Gender (M/F)	12/8	17/13	12/8	20/11
Full scale IQ (mean ± s.d.)^a^	111.1 ± 16.7	111.8 ± 14.5	72.2 ± 10.6	72.4 ± 11.5
DICA (*N*)	NA	NA	ADHD (6), Phobia (4), GAD (1), Enuresis (3), ODD(2)	ADHD (13), Phobia (6), GAD (3), Enuresis (1), Schizophrenia symptoms (2)
Antipsychotic treatment (*N*)	NA	NA	1	3
Antidepressant treatment (*N*)			1	5
Methylphenidate (*N*)			0	7

*TD* typically developing, *DICA* Diagnostic Interview for Children and Adolescents, *ADHD* attention deficit hyperactivity disorder, *ODD* oppositional defiant disorder, *MDD* major depressive disorder, *GAD* generalized anxiety disorder

^a^Full-scale IQ was significantly lower in 22q11.2 DS compared to typically developing participants (*t* = 14.6, d.f. = 89, *p* < 0.00001)

Normal hearing levels were reported for all participants. The participants were able to correctly discriminate the deviant stimuli from the standards in a brief behavioural test that followed the administration of the MMN paradigm.

### Neuropsychiatric and cognitive assessment

The participants' neuropsychiatric and cognitive profiles were evaluated by a trained psychiatrist (S.E.). Parents of 22q11.2 microdeletion carriers, children and adolescents under 18 years, were interviewed using the computerized Diagnostic Interview for Children and Adolescents-Revised (DICA)^56^ to identify the presence of psychiatric disorders in their children, while participants over 18 years old and their parents were interviewed using the Structured Clinical Interview for DSM-IV Axis I disorders^57^.

The participants were screened with the Wechsler Intelligence Scale for Children III-R (WISC-III-R)^58^ and the Wechsler Adult Intelligence Scale-III (WAIS-III; for participants >17 years)^59^. Information about full-scale IQ (FSIQ), performance IQ (PIQ), verbal IQ (VIQ), verbal comprehension (VCI), perceptual organization (POI), and processing speed were provided for all participants.

Psychotic symptoms were screened in 22q11.2 DS using the Structured Interview for Psychosis-Risk Syndromes (SIPS)^60^. The interview uses a 6-point severity scale (ranging from 0 to 6) to assess disorganization, general, negative, and positive symptoms. The assessment is based on the participants' answer, so children under 12 years old were not evaluated. A succinct description of prodromal symptoms of adolescents with 22q11.2 DS (as measured by the SIPS) is presented in Table 1.

### Stimuli and procedure

Sequences of auditory stimuli were presented binaurally using intra-aural insert earphones (Etymotic Research, USA) at an intensity of 65 dB SPL in one block of 600 tones. Standard stimuli (*N* = 480) were pure tones of 1000 Hz, while deviant stimuli (*N* = 120) were pure tones of 1200 Hz. The stimuli were randomly presented with a ratio of 8:2 via E-prime 1 (Psychology Software Tools Inc., Pittsburgh). The stimuli were 100 ms long (10 ms rise and fall) and were presented with an inter-stimulus interval (ISI) of 520 ± 2 ms.

Due to a problem with the presentation computer, some participants were presented with an ISI that varied between two values. In these cases, the ISI was either 507 ± 2 or 520 ms ± 2 ms long. This ISI variation was independent of group membership and occurred randomly within one run, affecting both standard and deviant stimuli.

In the cross-sectional study, 13 participants (3 typically developing and 10 22q11.2 deletion carriers) received in average 286/600 of the auditory stimuli with an ISI of 507 ± 2 ms instead of 520 ± 2 ms. In the longitudinal design, 13 participants (5 typically developing and 8 22q11.2 deletion carriers) received in average 276/600 of the auditory stimuli with a stimulus onset asynchrony of 507 ± 2 ms instead of 520 ± 5 ms. In addition, seven participants (four typically developing and three 22q11.2 deletion carriers) received the auditory stimuli with an ISI of 604 ± 2 ms.

Throughout stimulus presentation the participants were comfortably seated in a chair inside a Faraday shielded room. They were instructed to watch a silent cartoon movie (used as a visual distracter) on a monitor and to ignore the auditory events.

### Data acquisition

EEG data were continuously recorded with a sampling rate of 1000 Hz using a 256-electrodes Hydrocel cap (Electrical Geodesics Inc., Eugene, OR, USA), referenced to the vertex (Cz). Electrodes' impedance was monitored carefully and kept below 30 kΩ. For the reference electrode, the impedance was kept below 10 kΩ.

### Data pre-processing

For further analysis, the number of electrodes was reduced from 256 to 204 channels by eliminating electrodes on the cheek and the neck. The data were band-pass filtered between 1 and 40 Hz using non-causal Butterworth filters.

Independent Component Analysis (ICA) was applied to remove eye-movement (eye blinks and saccades) and ECG artefacts^61^ using a Matlab script based on the EEGlab runica function^62^ (https://sccn.ucsd.edu/eeglab/). After ICA artefact removal, peri-stimulus epochs between −100 to 450 ms were averaged for each participant separately for standard and deviant stimuli. The standards following a deviant and the deviants following less than two consecutive standards were rejected before averaging. Epochs with artefacts exceeding 60 µV were automatically excluded. In addition, the epochs were visually inspected and excluded if residual artefacts below these amplitude thresholds were detected.

In the longitudinal design, the accepted epochs did not differ significantly neither for the deviant stimulus (typically developing *t* = −1.4, d.f. = 13, *p* = 0.1, mean ± s.d. T1: 73.5 ± 7.1, mean ± s.d. T2: 74.7 ± 7.8; 22q11.2 DS group *t* = 1.1, d.f. = 15, *p* = 0.2, mean ± s.d. T1: 72.0 ± 8.4, mean ± s.d. T2: 71.7 ± 8.1) nor for the standard stimulus (typically developing *t* = 1, d.f. = 13, *p* = 0.3, mean ± s.d. T1: 246.1 ± 40.7, mean ± s.d. T2: 245.5 ± 40.4; 22q11.2 group *t* = 1.1, d.f. = 15, *p* = 0.2, mean ± s.d. T1: 241.3 ± 45.2, mean ± s.d. T2: 239.8 ± 46.1) between time point 1 and time point 2 of the recordings. In the cross-sectional study, the accepted epochs did not differ significantly between the four groups either for the deviant (F(3,97) = 0.45, *p* = 0.71) nor for the standard (F(3,97) = 0.48, *p* = 0.69). The mean ± s.d. of the accepted epochs is summarized in Table 3.

**Table 3:** Summary of the accepted epochs (cross-sectional study)

Epochs (mean ± s.d.)	TD participants children	TD participants adolescents	22q11.2DS children	22q11.2DS adolescents
Deviant	81.65 ± 8.0	79.56 ± 10.1	81.85 ± 7.6	79.7 ± 8.7
Standard	276.65 ± 45.5	263.96 ± 46.6	263.95 ± 46.4	261.77 ± 44.4

*TD* typically developing

No baseline correction was applied, since we compare difference waves across groups. Noisy channels were interpolated using a spherical spline interpolation^63^. Averaged data were recalculated from vertex reference to the common average reference. Mismatch responses were then individually calculated by subtracting each standard evoked potential from each deviant evoked potential.

These pre-processing steps were performed using Cartool software: https://sites.google.com/site/cartoolcommunity/

### Data analyses

To statistically investigate the longitudinal changes in amplitude within the deletion carriers and the typically developing group, the following analyses were applied on the difference waveforms.

First, an exploratory analysis of all electrodes, time point by time point between 0 and 300 ms post-stimulation using paired *t*-tests for amplitude differences, was performed. A significance level of *p* < 0.05 and a temporal constraint of 20 ms (tf; 20 ms within which the significance threshold needed to be maintained) were fixed. To overcome the multiple comparison problem, due to multiple sensors and multiple time points, we additionally ran non-parametric permutation tests^64^, using the same significance level of *p* < 0.05 and the same temporal constraint of 20 ms (20 tf) of significance. To quantify the size of the difference between the groups, the mean amplitude around the MMN peak (selected from the group average waveforms) was calculated for each individual over the fronto-central cluster of channels that showed significant differences in the exploratory analysis. Paired *t*-tests (*p*-values <0.05) and Cohen’s *d* effect size^65^ for dependent samples were computed.

Topographic differences of scalp potential maps were quantified using the Global Map Dissimilarity (GMD), an index of configuration dissimilarities between two electric fields at a given point in time. The GMD is an individual measure of the distance between two vectors or two electric field topographies, both normalized to unitary global field power. It is equivalent to the spatial Pearson’s product-moment correlation coefficient between two adjacent topographic maps^66^. Map dissimilarities were statistically assessed using a paired topographical bootstrapping approach: the topographic analysis of variance (TANOVA). It is important to clarify that the TANOVA is based on a non-parametric randomization approach and not an analysis of variance, as the given name of this analysis might suggest^67^. To statistically investigate topographic differences by paired TANOVAs, a significance level of *p* < 0.05 and a temporal criterion of 20 ms of significance (tf 20 ms) was applied.

To identify differences in amplitude for the MMN response between the typically developing groups and the 22q11.2 deletion carriers, we applied the same pipeline as described for the longitudinal study using unpaired two-tailed *t*-tests for equal variance (Levene F(1,59) = 0.95 *p* = 0.33 for the adolescents' groups; Levene F(1,38) = 3.38 *p* = 0.07 for the children’groups) and unpaired non-parametric permutation tests for waveform comparisons and unpaired TANOVAs for topographic differences. The statistical analyses were performed using the Cartool software (https://sites.google.com/site/fbmlab/cartool) and Statistica 13 (TIBCO Software Inc).

### Correlations with clinical data

In the longitudinal design, we performed a Spearman’s rank correlation coefficient analysis using Statistica software to evaluate the linear relationship between the MMN response and cognitive function (full-scale, performance and verbal IQ using WISC-III-R or WAIS-III for participants >17 years old). We also performed a Spearman’s rank correlation coefficient analysis to evaluate the linear relationship between the MMN response and the clinical symptom scores (SIPS) for the 22q11.2 deletion carriers.

For this purpose, we computed the mean amplitude over a cluster of 16 fronto-central channels (E6, E7, E8, E9 E14, Fcz, E16, E17, Fz, E22, E23, Fc1, Fc2, E186, E198, Cz). We identified the peak of the MMN component in the group averaged difference waveforms. We considered the 16 fronto-central channels and we calculated the mean over 30 ms around the peak, 155–185 ms post-stimulus, for each individual. The mean amplitude was used for the correlation analysis with the clinical and cognitive (IQ) data. The correlations were considered significant for *p-*values <0.01.

## Results

### Longitudinal study

The 22q11.2 deletion carriers show a reduced MMN response from childhood to adolescence. Paired *t*-tests show a significant decrease in mean amplitude for the MMN response over the fronto-central channels (*t* = −3.51, d.f. = 15, *p* = 0.003) with a large effect size (*d* = −0.85) between T1 and T2. Furthermore, we observe a significant change in the topographic distribution from childhood to adolescence (topographical bootstrapping approach TANOVA; *p* < 0.05; tf 20 ms). The results of the paired *t*-tests over all the electrodes across all time points were confirmed by non-parametrical statistical testing.

**Fig. 1:**
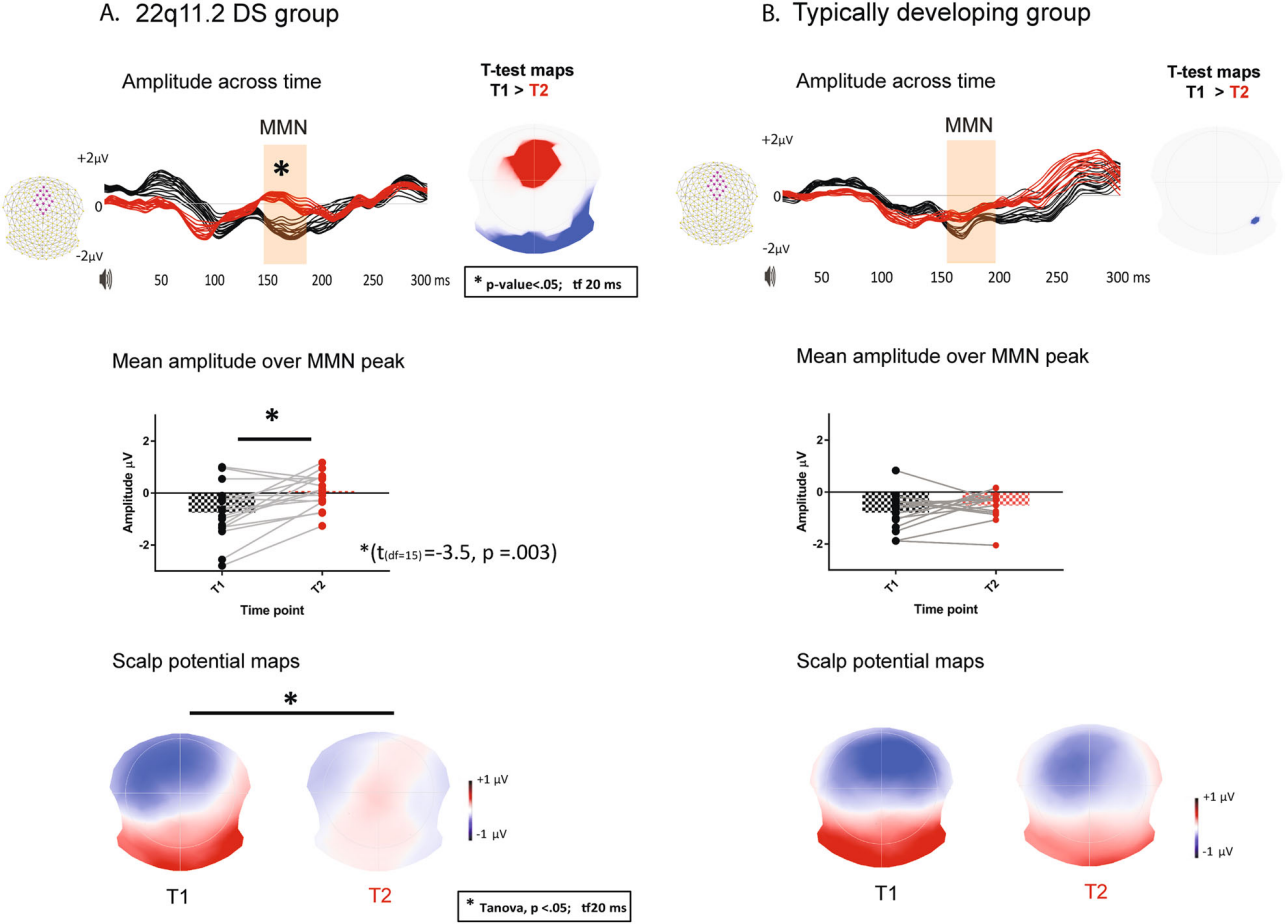
Difference waveform analyses in the longitudinal design The 22q11.2DS group is plotted on the left side, while the typically developing (TD) group on the right side. Mean amplitude across time: the amplitude over a cluster of fronto-central channels (displayed in pink) is plotted over time (red for adolescents, black for children). Alongside, the topographic map of the *t*-test is showing the channels with significant *p*-values (the positive values in red indicate significantly higher negative amplitudes for children compared to adolescents; the negative values in blue indicate significantly higher positive amplitudes for children compared to adolescents). Mean amplitude over the MMN peak: a scatter plot distribution of the mean amplitudes measured at the fronto-central cluster of electrodes over 30 ms around the MMN peak (155–185 ms; black during childhood or time point 1 and red during adolescence or time point 2). The scalp potential maps represent the topographical distribution as potential maps of the mismatch negativity response over 155–185 ms post-stimulus

No significant differences in mean amplitude or topographic distribution are seen within the typically developing group from childhood to adolescence (*t* = −1.32, d.f. = 13, *p* = 0.20). The results are presented in Fig. 1.

In addition, the paired *t*-tests between adolescents and children with 22q11.2 deletion reveal a significantly increased amplitude for an earlier component, the N1 (75–100 ms post-stimulus) over the fronto-central channels (paired two-tailed *t*-test; *p* < 0.05; tf 20 ms) during adolescence. We observe a significant change in topographic distribution from childhood to adolescence also for the N1 component, in addition to the changes responsible for the mismatch response (topographical bootstrapping approach; *p* < 0.05; tf 20 ms).

### Correlations with clinical data

The Spearman’s rank correlation coefficient did not reveal any significant correlations between the mean amplitude, cognitive (IQ), and clinical scores (SIPS).

In the longitudinal study, the full scale IQ did not significantly differ in the 22q11.2 deletion carriers (*t* = −0.5, d.f. = 14, *p*-value = 0.5) or the typically developing group (*t* = −1.1, d.f. = 10, *p*-value = 0.2) between T1 and T2. Nevertheless, in the cross-sectional study, the full-scale IQ was significantly lower in 22q11.2 DS compared to typically developing participants (*t* = 14.6, d.f. = 89, *p* < 0.00001).

In the longitudinal subgroup of patients, the severity of the prodromal symptoms (data available only for 12 participants) did not differ between T1 and T2 (disorganization *t* = −0.41, d.f. = 11, *p* = 0.68; general *t* = −0.92, d.f. = 11, *p* = 0.37; negative *t* = −1.62, d.f. = 11, *p* = 0.13; positive *t* = 2.15, d.f. = 11, *p* = 0.05).

### Cross-sectional study

The cross-sectional design reveals also a significantly reduced MMN response in adolescents with 22q11.2 DS.

In comparison with the typically developing adolescents, the 22q11.2 deletion carriers show reduced amplitude over the central electrodes (slightly lateralized on the left side; unpaired two-tailed *t*-test; p < 0.05; tf 20 ms) within the time window of the MMN (150–180 ms post-stimulus). The results of unpaired, two-tailed *t*-tests were confirmed by non-parametrical statistical testing. After calculating the mean amplitude (30 ms over the central electrodes), the results were significant (*t* = −2.9, d.f. = 59, *p* = 0.005) with a medium effect size (*d* = −0.75). However, no statistically significant topographic changes were observed between the two adolescent groups (topographical bootstrapping approach; *p* < 0.05; tf 20 ms).

**Fig. 2:**
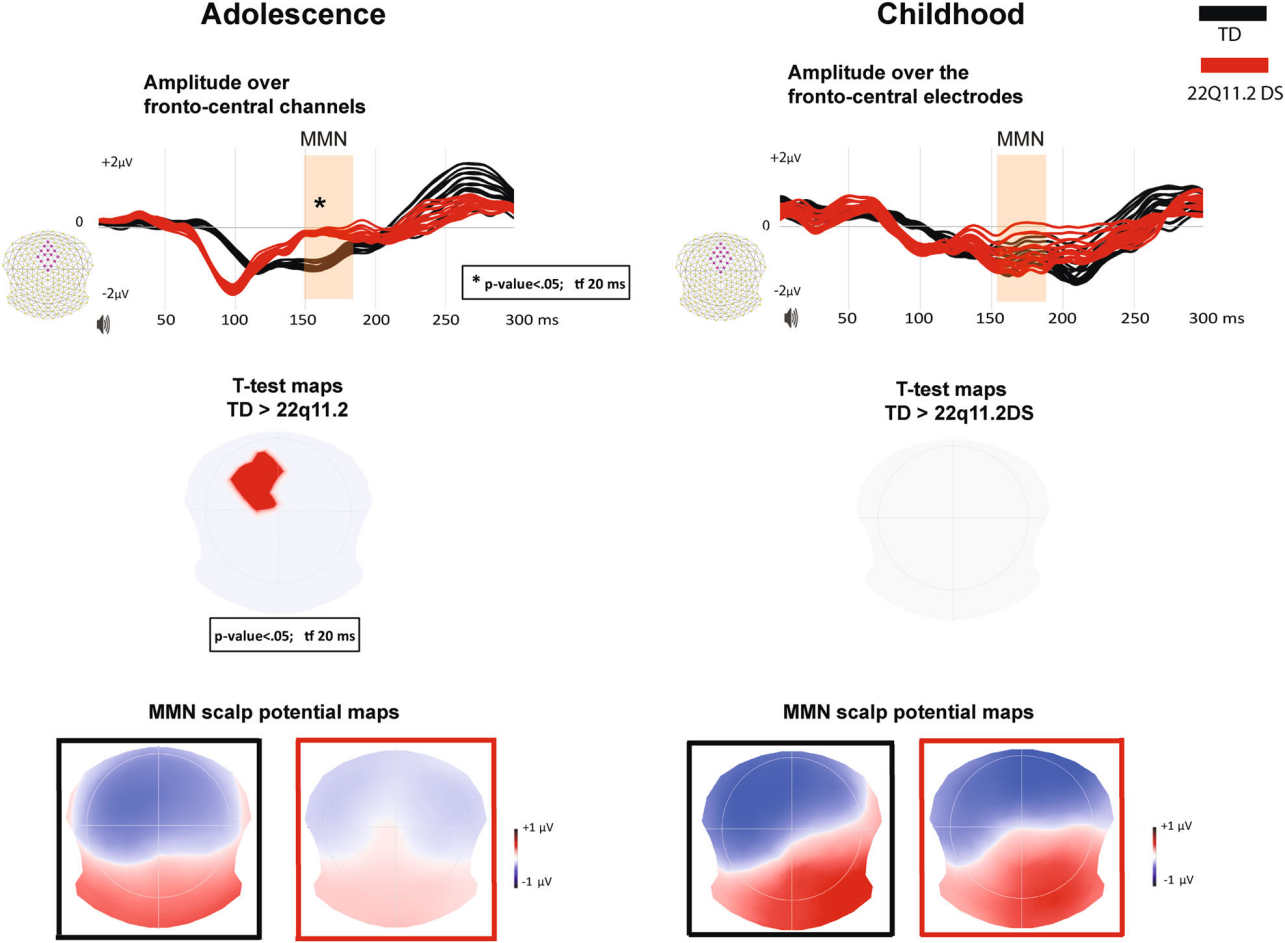
Difference waveform analyses in the cross-sectional design. The adolescents are plotted on the left side, while the younger groups are on the right side. Mean amplitude over a cluster of fronto-central channels (displayed in pink on the left side) is plotted over time (red for 22q11.2 carriers, black for typically developing individuals (TD)). The topographic map of the *t*-test is showing the channels with significant *p*-values (the positive values in red indicate significantly higher negative amplitudes for TD adolescents over the central channels compared to adolescents with 22q11.2 deletion). The scalp potential maps of the mismatch negativity response over 150–200 ms post-stimulus are shown below

No significant differences in amplitude (*t* = −0.56, d.f. = 38, *p* = 0.57) or topographic distribution are seen when comparing the two groups of children, typically developing and 22q11.2 deletion carriers. The results are presented in Fig. 2.

Furthermore, the adolescents with 22q11.2DS show a significantly increased amplitude during the time window of the N1 component over fronto-central channels (80–100 ms post-stimulus) and a reduced amplitude during the P3 component (250–300 ms post-stimulus) over the central channels (unpaired two-tailed *t*-test; *p* < 0.05; tf 20 ms) compared to the typically developing adolescents.

## Discussion

The MMN decrease in amplitude is a robust neurophysiological dysfunction in subjects with schizophrenia^33,34^ and in young individuals at high-risk of developing this disorder^32^. It might be explained by grey matter volume reduction, impaired connectivity, and dysfunctional cortical glutamate *N*-methyl-d-aspartate receptors (NMDAr) in the frontal and temporal cortices^45,68,69^.

The results from both the longitudinal and cross-sectional studies reveal the emergence of schizophrenia-like early auditory sensory processing deficits during adolescence, a period of considerable brain changes and a vulnerable window for the emergence of psychotic disorders^70–73^.

In the longitudinal design, the 22q11.2 deletion carriers exhibit a reduction in amplitude and a change in topographic patterns of the MMN response from childhood to adolescence. The altered mismatch response in adolescents with 22q11.2DS is also revealed by the cross-sectional approach.

By law of physics, topographic differences of the scalp potential maps can be interpreted as changes in the cortical activation pattern^74,75^. In the developing brain, these changes may be due to dissimilarities in dipole orientation resulting from changes in cortical folding with age^76^. Further, they may be due to changes in functional organization of the maturing brain, meaning that the neural sources recruited during the task may engage differently across stages of development, as a consequence of structural brain maturation^77^.

It is well established in 22q11.2 deletion carriers that from childhood to adolescence the brain is fine-tuning its architecture differently compared to the typically developing population^70,78^. The age-related cortical thinning in the typically developing subjects initiates in childhood in the primary sensorimotor areas, spreads rostrally over the frontal cortex, then caudally and laterally over the parietal, occipital, and lastly the temporal cortex^79^. In 22q1.2 DS, a different developmental pattern is observed. In a longitudinal study, Schaer et al.^80^ report subtle cortical thickening during childhood predominantly in the prefrontal cortex and increased cortical loss over widespread clusters starting with adolescence.

In addition, aberrant brain connectivity in adolescence has also been reported in humans^81,82^ and animal models of 22q11.2 DS^17^. Ottet et al.^82^ describe reduced left fronto-temporal connections and increased right fronto-frontal connections in patients with 22q11.2 DS compared with typically developing participants.

In animal models, Chun et al.^17^ find disruptions in the activity of thalamo-cortical glutamatergic projections to the auditory cortex, that is becoming evident only after 3 months of age in a mouse model of 22q11.2DS (corresponding to early adulthood in humans).

The frequency deviant mismatch response (fMMN) has been proposed to rely mostly on the activation of the “lemniscal areas” or core thalamo-cortical projections, which carry tonotopically organized and auditory specific information from the ventral medial geniculate nuclei to the primary auditory cortex^37,83,84^. In line with these results, further evidence indicates that in humans the 22q11.2 deletion is associated with reduced thalamic volume, prominently in the posterior region^85^ and reduced auditory cortex surface area^2^. These results might suggest that the normal flow of auditory information is impaired at the subcortical level and deficits in the “core” thalamo-cortical pathway could partially explain the pathogenic mechanisms that mediate fMMN impairments in 22q11.2 deletion carriers.

Consequently, the attenuated mismatch response among the 22q11.2 deletion carriers may result from progressive cortical loss and volumetric reduction in the MGN, frontal (inferior prefrontal and medial frontal gyri) and temporal cortices (superior temporal cortex, AC)^15,86–89^, impaired connectivity^82^, and dysfunctional glutamate *N*-methyl-d-aspartate receptors (NMDAr)^90,91^ within these areas.

It is thus possible that the MMN response is sensitive to subtle structural changes in cortical and thalamic areas, reflecting aberrant brain maturation in 22q11.2 DS.

Other studies did not report significant MMN reduction in adolescents with 22q11.2 DS in response to the frequency deviant^46,48^. The inconsistency between these findings and those reported by Baker et al.^48^ might be due to the fact that the authors report the amplitude over the Fz channel, while a larger cluster of significant fronto-central channels is considered in the present study. Nevertheless, the authors found a reduction in the response to duration deviants. The dMMN was proposed as an index trait marker of schizophrenia, whereas the fMMN may be related to progressive brain pathology related to the disorder^37,55^. Therefore, it is important to highlight the need of adding the dMMN deviant in future investigations.

Further, the inconsistency with the results observed in the Larsen et al. study^46^ might also highlight the phenotype heterogeneity of 22q11.2 deletion carriers. The authors report an altered functional connectivity from IFG to STG that did not coincide with an amplitude reduction. These results, even though they did not survive multiple comparison correction, might be informative and complementary to ours by adding the connectivity information and highlight the imperious need for further investigation.

Additionally, we did not find significant correlations between the prodromal symptoms, positive or negative, measured with SIPS and the mismatch response attenuation and we did not observe a significant change in the intensity of symptoms from T1 to T2.

These results go in line with the literature showing no consistent relationships between MMN size and the severity of psychotic symptoms^92^.

Likewise, we speculate that the fMMN attenuation might be tied to an increased vulnerability to develop schizophrenia-like symptoms later in life, but it may also be intimately linked to abnormal cortical development without compulsory transition into a schizophrenic state.

In typically developing participants, we observe no significant change in the MMN response, in either amplitude or topography, corroborating the hypothesis that typically developing subjects do not show robust changes of the mismatch response from childhood to adolescence. This effect is in keeping with previous MMN studies demonstrating that this component is developmentally quite stable in terms of amplitude^53,93^, but disagree with the studies that find significant MMN amplitude decreases^52^ or increases^51^ with age. Nevertheless, several studies suggest that the MMN response results from brain maturational processes across ages even when it seems stable in amplitude across development^94–97^.

A strong effect highlighted by both, the longitudinal and cross-sectional studies, is the significant increase in amplitude and changes in topographic distribution of the auditory N1 component during adolescence in 22q11.2 deletion carriers. This observation confirms the results published by our group in a study which showed clear alteration for the N1 component in adolescents with 22q11.2 DS compared with typically developing participants^14^. Using a different auditory passive paradigm (P50, a paired click test) the authors found an increase in early, central N1 and a decrease in the second, lateral N1 in adolescents with 22q11DS. The alterations were explained by elevated activity in the anterior cingulate and dorsomedial frontal cortex followed by a diminution in activity in left superior temporal gyrus in 22q11.2 DS.

Further, the increase in N1 amplitude might also point towards alterations in the cortical glutamate *N*-methyl-d-aspartate receptors (NMDAr). As an effect of ketamine administration (NMDAr antagonist) in typically developing subjects, Oranje et al.^98^ and Umbricht et al.^42^ reported an enhanced N1 response to the deviant stimuli. Additionally, in the cross-sectional study, we observe a decrease in the P3 component in adolescents with 22q11.2 DS. The same effect is reported in patients with schizophrenia and also by studies testing the effect of ketamine (NMDAr antagonist) on typically developing subjects^99^.

It is important to note some limitations of the current study. First, our sample of 22q11.2 deletion carriers expresses heterogeneous levels of psychosis risk and medication status. Second, we had an ISI difference due to a problem with the presentation computer during a brief period. Nevertheless, the ISI varied within the same paradigm of MMN presentation and the ISI difference was very short (<15 ms) and was equally affecting the groups. Third, we did not include a duration deviant. This might be relevant for future studies, as deviant types might produce specific biomarkers for functional levels in patients with schizophrenia and individuals at risk^37,55^.

In summary, we observe auditory neurophysiological abnormalities in non-psychotic 22q11DS adolescents similar to those found in schizophrenia. The auditory processing impairments might be promoted by two main effects: local structural and molecular brain alterations and abnormal interactions between the auditory brain areas, and might co-occur with the increased risk to develop schizophrenia-like symptoms later in life. In this view, future work should explore the link between cortical changes, functional connectivity, glutamate dysfunction, and the variability of the MMN response, both for fMMN and dMMN, across ages in the 22q11.2 DS population with respect to the severity of prodromal symptoms.

## Data Availability

The datasets generated and analysed during the current study are available from the corresponding author on reasonable request.

## References

[1] Karayiorgou M, Simon TJ, Gogos JA (2010). 22q11.2 microdeletions: linking DNA structural variation to brain dysfunction and schizophrenia. Nat. Rev. Neurosci..

[2] Lin A (2017). Mapping 22q11.2 gene dosage effects on brain morphometry. J. Neurosci..

[3] Jonas RK, Montojo CA, Bearden CE (2014). The 22q11.2 deletion syndrome as a window into complex neuropsychiatric disorders over the lifespan. Biol. Psychiatry.

[4] Green T (2009). Psychiatric disorders and intellectual functioning throughout development in velocardiofacial (22q11.2 deletion) syndrome. J. Am. Acad. Child Adolesc. Psychiatry.

[5] Schneider M (2014). Clinical and cognitive risk factors for psychotic symptoms in 22q11.2 deletion syndrome: a transversal and longitudinal approach. Eur. Child Adolesc. Psychiatry.

[6] Gur RE (2017). A neurogenetic model for the study of schizophrenia spectrum disorders: the International 22q11.2 Deletion Syndrome Brain Behavior Consortium. Mol. Psychiatry.

[7] Tang SX (2017). Emergent, remitted and persistent psychosis-spectrum symptoms in 22q11.2 deletion syndrome. Transl. Psychiatry.

[8] Devriendt K, Fryns JP, Mortier G, van Thienen MN, Keymolen K (1998). The annual incidence of DiGeorge/velocardiofacial syndrome. J. Med. Genet..

[9] Scambler PJ (2000). The 22q11 deletion syndromes. Hum. Mol. Genet..

[10] Meechan DW, Tucker ES, Maynard TM, LaMantia AS (2009). Diminished dosage of 22q11 genes disrupts neurogenesis and cortical development in a mouse model of 22q11 deletion/DiGeorge syndrome. Proc. Natl. Acad. Sci. USA.

[11] Swillen A, Moss E, Duijff S (2018). Neurodevelopmental outcome in 22q11.2 deletion syndrome and management. Am. J. Med. Genet..

[12] Wong LM, Riggins T, Harvey D, Cabaral M, Simon TJ (2014). Children with chromosome 22q11.2 deletion syndrome exhibit impaired spatial working memory. Am. J. Intellect. Dev. Disabil..

[13] Biria M (2018). Visual processing deficits in 22q11.2 deletion syndrome. NeuroImage Clin..

[14] Rihs TA (2013). Altered auditory processing in frontal and left temporal cortex in 22q11.2 deletion syndrome: a group at high genetic risk for schizophrenia. Psychiatry Res..

[15] Gothelf D, Schaer M, Eliez S (2008). Genes, brain development and psychiatric phenotypes in velo-cardio-facial syndrome. Dev. Disabil. Res. Rev..

[16] Van L, Boot E, Bassett AS (2017). Update on the 22q11.2 deletion syndrome and its relevance to schizophrenia. Curr. Opin. Psychiatry.

[17] Chun S (2017). Thalamic miR-338-3p mediates auditory thalamocortical disruption and its late onset in models of 22q11.2 microdeletion. Nat. Med..

[18] Turetsky BI (2007). Neurophysiological endophenotypes of schizophrenia: the viability of selected candidate measures. Schizophr. Bull..

[19] Mantysalo S, Naatanen R (1987). The duration of a neuronal trace of an auditory stimulus as indicated by event-related potentials. Biol. Psychol..

[20] Naatanen R, Paavilainen P, Rinne T, Alho K (2007). The mismatch negativity (MMN) in basic research of central auditory processing: a review. Clin. Neurophysiol..

[21] Garrido MI, Kilner JM, Stephan KE, Friston KJ (2009). The mismatch negativity: a review of underlying mechanisms. Clin. Neurophysiol..

[22] Paavilainen P (2013). The mismatch-negativity (MMN) component of the auditory event-related potential to violations of abstract regularities: a review. Int. J. Psychophysiol..

[23] Giard MH, Perrin F, Pernier J, Bouchet P (1990). Brain generators implicated in the processing of auditory stimulus deviance: a topographic event-related potential study. Psychophysiology.

[24] Rosburg T (2005). Subdural recordings of the mismatch negativity (MMN) in patients with focal epilepsy. Brain.

[25] El Karoui I (2015). Event-related potential, time-frequency, and functional connectivity facets of local and global auditory novelty processing: an intracranial study in humans. Cereb. Cortex.

[26] Molholm S, Martinez A, Ritter W, Javitt DC, Foxe JJ (2005). The neural circuitry of pre-attentive auditory change-detection: an fMRI study of pitch and duration mismatch negativity generators. Cereb. Cortex.

[27] Doeller CF (2003). Prefrontal cortex involvement in preattentive auditory deviance detection: neuroimaging and electrophysiological evidence. Neuroimage.

[28] Opitz B, Rinne T, Mecklinger A, von Cramon DY, Schroger E (2002). Differential contribution of frontal and temporal cortices to auditory change detection: fMRI and ERP results. Neuroimage.

[29] Gaebler AJ (2015). Auditory mismatch impairments are characterized by core neural dysfunctions in schizophrenia. Brain.

[30] Duncan CC (2009). Event-related potentials in clinical research: guidelines for eliciting, recording, and quantifying mismatch negativity, P300, and N400. Clin. Neurophysiol..

[31] Nagai T (2013). Mismatch negativity as a “translatable” brain marker toward early intervention for psychosis: a review. Front. Psychiatry.

[32] Lavoie S (2018). Impaired mismatch negativity to frequency deviants in individuals at ultra-high risk for psychosis, and preliminary evidence for further impairment with transition to psychosis. Schizophr. Res..

[33] Umbricht D, Krljes S (2005). Mismatch negativity in schizophrenia: a meta-analysis. Schizophr. Res..

[34] Erickson MA, Ruffle A, Gold JM (2016). A meta-analysis of mismatch negativity in schizophrenia: from clinical risk to disease specificity and progression. Biol. Psychiatry.

[35] Todd J, Harms L, Michie P, Schall U (2013). Mismatch negativity: translating the potential. Front. Psychiatry.

[36] Avissar M (2018). Meta-analysis of mismatch negativity to simple versus complex deviants in schizophrenia. Schizophr. Res..

[37] Lee M (2018). A tale of two sites: differential impairment of frequency and duration mismatch negativity across a primarily inpatient versus a primarily outpatient site in schizophrenia. Schizophr. Res..

[38] Rasser PE (2011). Gray matter deficits, mismatch negativity, and outcomes in schizophrenia. Schizophr. Bull..

[39] Sweet RA, Henteleff RA, Zhang W, Sampson AR, Lewis DA (2009). Reduced dendritic spine density in auditory cortex of subjects with schizophrenia. Neuropsychopharmacologyy.

[40] Moghaddam B, Javitt D (2012). From revolution to evolution: the glutamate hypothesis of schizophrenia and its implication for treatment. Neuropsychopharmacology.

[41] Javitt DC, Steinschneider M, Schroeder CE, Arezzo JC (1996). Role of cortical N-methyl-D-aspartate receptors in auditory sensory memory and mismatch negativity generation: implications for schizophrenia. Proc. Natl. Acad. Sci. USA.

[42] Umbricht D (2000). Ketamine-induced deficits in auditory and visual context-dependent processing in healthy volunteers: implications for models of cognitive deficits in schizophrenia. Arch. Gen. Psychiatry.

[43] Lavoie S (2008). Glutathione precursor, N-acetyl-cysteine, improves mismatch negativity in schizophrenia patients. Neuropsychopharmacology.

[44] Kantrowitz JT (2018). Improvement in mismatch negativity generation during d-serine treatment in schizophrenia: correlation with symptoms. Schizophr. Res..

[45] Catts VS, Lai YL, Weickert CS, Weickert TW, Catts SV (2016). A quantitative review of the postmortem evidence for decreased cortical N-methyl-d-aspartate receptor expression levels in schizophrenia: how can we link molecular abnormalities to mismatch negativity deficits?. Biol. Psychol..

[46] Larsen KM (2018). Altered auditory processing and effective connectivity in 22q11.2 deletion syndrome. Schizophr. Res..

[47] Zarchi O (2013). Schizophrenia-like neurophysiological abnormalities in 22q11.2 deletion syndrome and their association to COMT and PRODH genotypes. J. Psychiatr. Res..

[48] Baker K, Baldeweg T, Sivagnanasundaram S, Scambler P, Skuse D (2005). COMT Val108/158 Met modifies mismatch negativity and cognitive function in 22q11 deletion syndrome. Biol. Psychiatry.

[49] Cheour M (1998). Mismatch negativity (MMN) as an index of auditory sensory memory deficit in cleft-palate and CATCH syndrome children. Neuroreport.

[50] Oades RD, Dmittmann-Balcar A, Zerbin D (1997). Development and topography of auditory event-related potentials (ERPs): mismatch and processing negativity in individuals 8–22 years of age. Psychophysiology.

[51] Bishop DVM, Hardiman MJ, Barry JG (2011). Is auditory discrimination mature by middle childhood? A study using time-frequency analysis of mismatch responses from 7 years to adulthood. Dev. Sci..

[52] Shafer VL, Morr ML, Kreuzer JA, Kurtzberg D (2000). Maturation of mismatch negativity in school-age children. Ear. Hear..

[53] Mahajan Y, McArthur G (2015). Maturation of mismatch negativity and P3a response across adolescence. Neurosci. Lett..

[54] Cheng CH, Hsu WY, Lin YY (2013). Effects of physiological aging on mismatch negativity: a meta-analysis. Int. J. Psychophysiol..

[55] Näätänen R, Shiga T, Asano S, Yabe H (2015). Mismatch negativity (MMN) deficiency: a break-through biomarker in predicting psychosis onset. Int. J. Psychophysiol..

[56] Reich W (2000). Diagnostic Interview for Children and Adolescents (DICA). J. Am. Acad. Child Adolesc. Psychiatry.

[57] First, M. B. & Gibbon, M. in *Comprehensive Handbook of Psychological Assessment, Vol. 2: Personality Assessment* (ed. Hersen, M.) 134–143 (John Wiley & Sons Inc., Hoboken, New Jersey, 2004).

[58] Franzen, M. D. in *Reliability and Validity in Neuropsychological Assessment* (ed. Franzen, M. D.) 71–89 (Springer Science +Business Media, New York, 2002).

[59] Wechsler, D. *Manual for the Wechsler Adult Intelligence Scale*. (Psychological Corp., Oxford, England, 1955).

[60] Miller TJ (2003). Prodromal assessment with the structured interview for prodromal syndromes and the scale of prodromal symptoms: predictive validity, interrater reliability, and training to reliability. Schizophr. Bull..

[61] Jung TP (2000). Removal of eye activity artifacts from visual event-related potentials in normal and clinical subjects. Clin. Neurophysiol..

[62] Makeig S, Jung TP, Bell AJ, Ghahremani D, Sejnowski TJ (1997). Blind separation of auditory event-related brain responses into independent components. Proc. Natl. Acad. Sci. USA.

[63] Perrin F, Pernier J, Bertrand O, Echallier JF (1989). Spherical splines for scalp potential and current density mapping. Electroencephalogr. Clin. Neurophysiol..

[64] Maris E, Oostenveld R (2007). Nonparametric statistical testing of EEG- and MEG-data. J. Neurosci. Methods.

[65] Cohen J (1992). A power primer. Psychol. Bull..

[66] Murray MM, Brunet D, Michel CM (2008). Topographic ERP analyses: a step-by-step tutorial review. Brain Topogr..

[67] Habermann M, Weusmann D, Stein M, Koenig T (2018). A student’s guide to randomization statistics for multichannel event-related potentials using Ragu. Front. Neurosci..

[68] Weickert CS (2013). Molecular evidence of N-methyl-D-aspartate receptor hypofunction in schizophrenia. Mol. Psychiatry.

[69] Takahashi T (2009). Progressive gray matter reduction of the superior temporal gyrus during transition to psychosis. Arch. Gen. Psychiatry.

[70] Paus T (2005). Mapping brain maturation and cognitive development during adolescence. Trends Cogn. Sci..

[71] Giedd JN, Keshavan M, Paus T (2008). Why do many psychiatric disorders emerge during adolescence?. Nat. Rev. Neurosci..

[72] Galvan A (2017). Adolescence, brain maturation and mental health. Nat. Neurosci..

[73] Casey BJ, Getz S, Galvan A (2008). The adolescent brain. Dev. Rev..

[74] Vaughan HG (1982). The neural origins of human event-related potentials. Ann. NY Acad. Sci..

[75] Michel, C. M., He, B. in *Niedermeyer’s Electroencephalography: Basic Principles, Clinical Applications, and Related Fields Seventh Edition* (eds. Schomer, D. L. & Lopes da Silva, F. H.) Chap. 45, 1135–1156 (Oxford University Press, New York, 2018).

[76] Cao B (2017). Lifespan gyrification trajectories of human brain in healthy individuals and patients with major psychiatric disorders. Sci. Rep..

[77] Casey BJ, Tottenham N, Liston C, Durston S (2005). Imaging the developing brain: what have we learned about cognitive development?. Trends Cogn. Sci..

[78] Tamnes CK (2010). Brain maturation in adolescence and young adulthood: regional age-related changes in cortical thickness and white matter volume and microstructure. Cereb. Cortex.

[79] Gogtay N (2004). Dynamic mapping of human cortical development during childhood through early adulthood. Proc. Natl. Acad. Sci. USA.

[80] Schaer M (2009). Deviant trajectories of cortical maturation in 22q11.2 deletion syndrome (22q11DS): a cross-sectional and longitudinal study. Schizophr. Res..

[81] Sandini C (2018). Development of structural covariance from childhood to adolescence: a longitudinal study in 22q11.2DS. Front. Neurosci..

[82] Ottet MC (2013). Reduced fronto-temporal and limbic connectivity in the 22q11.2 deletion syndrome: vulnerability markers for developing schizophrenia?. PLoS ONE.

[83] Jones EG (2009). Synchrony in the interconnected circuitry of the thalamus and cerebral cortex. Ann. NY Acad. Sci..

[84] Lee M (2017). Neural mechanisms of mismatch negativity dysfunction in schizophrenia. Mol. Psychiatry.

[85] Bish JP, Nguyen V, Ding L, Ferrante S, Simon TJ (2004). Thalamic reductions in children with chromosome 22q11.2 deletion syndrome. Neuroreport.

[86] Zinkstok J, van Amelsvoort T (2005). Neuropsychological profile and neuroimaging in patients with 22Q11.2 Deletion Syndrome: a review. Child Neuropsychol..

[87] Bearden CE (2007). Mapping cortical thickness in children with 22q11.2 deletions. Cereb. Cortex.

[88] Bearden CE (2009). Alterations in midline cortical thickness and gyrification patterns mapped in children with 22q11.2 deletions. Cereb. Cortex.

[89] Sun, D. et al. Large-scale mapping of cortical alterations in 22q11.2 deletion syndrome: convergence with idiopathic psychosis and effects of deletion size. *Mol. Psychiatry* (2018) 10.1038/s41380-018-0078-5. 10.1038/s41380-018-0078-5 PMC6292748 29895892

[90] da Silva Alves F (2011). Proton magnetic resonance spectroscopy in 22q11 deletion syndrome. PLoS ONE.

[91] Evers LJM (2015). Glutamatergic markers, age, intellectual functioning and psychosis in 22q11 deletion syndrome. Psychopharmacol. (Berl.).

[92] Erickson M, Ruffle A, Fleming L, Corlett P, Gold J (2017). 49. The predictive coding account of psychosis: a meta-analysis of the relationship between mismatch negativity and symptom severity. Schizophr. Bull..

[93] Cheour M, Leppanen PH, Kraus N (2000). Mismatch negativity (MMN) as a tool for investigating auditory discrimination and sensory memory in infants and children. Clin. Neurophysiol..

[94] Gomot M, Giard MH, Roux S, Barthelemy C, Bruneau N (2000). Maturation of frontal and temporal components of mismatch negativity (MMN) in children. Neuroreport.

[95] Morr ML, Shafer VL, Kreuzer JA, Kurtzberg D (2002). Maturation of mismatch negativity in typically developing infants and preschool children. Ear. Hear..

[96] Martin BA, Shafer VL, Morr ML, Kreuzer JA, Kurtzberg D (2003). Maturation of mismatch negativity: a scalp current density analysis. Ear. Hear..

[97] Cooray GK, Garrido MI, Brismar T, Hyllienmark L (2016). The maturation of mismatch negativity networks in normal adolescence. Clin. Neurophysiol..

[98] Oranje B (2000). The effects of a sub-anaesthetic dose of ketamine on human selective attention. Neuropsychopharmacology.

[99] Schwertner A, Zortea M, Torres FV, Caumo W (2018). Effects of subanesthetic ketamine administration on visual and auditory event-related potentials (ERP) in humans: a systematic review. Front. Behav. Neurosci..

